# The Effects of Voriconazole on Metabolism of All-Trans Retinoic Acid in the Treatment of Acute Promyelocytic Leukemia: A Case Report

**DOI:** 10.7759/cureus.13337

**Published:** 2021-02-14

**Authors:** Yusra Hashmi, Shehzeen F Memon, Yasir A Khan, Naeem Jabbar, Neelum Mansoor

**Affiliations:** 1 Pediatric Oncology, The Indus Hospital, Karachi, PAK; 2 Internal Medicine, Dow University of Health Sciences, Karachi, PAK; 3 Internal Medicine, Morriston Hospital, Swansea, GBR; 4 Hematology and Oncology, The Indus Hospital, Karachi, PAK

**Keywords:** all-trans retinoic acid, acute promyelocytic leukemia, voriconazole, hypercalcemia

## Abstract

All-trans retinoic acid (ATRA) is a derivative of vitamin A and is the mainstay treatment of acute promyelocytic leukemia (APL). Hypercalcemia is a rare yet important side-effect of ATRA, especially when it is used concomitantly with a medication that impedes its metabolism by inhibiting cytochrome P-450 in the liver and thus increasing the duration of exposure to ATRA. Azole antifungal drugs such as voriconazole are frequently used in patients undergoing chemotherapy due to a high incidence of fungal infections. These medications inhibit two vital enzymes of cytochrome P-450, CYP2C9 and CYP3A4, potentiating the effects of ATRA on calcium metabolism. We present a case of a nine-year-old girl who underwent chemotherapy with all-trans retinoic acid for acute promyelocytic leukemia. The patient was given an anti-fungal cover with voriconazole for extensive fungal chest infection simultaneously. She was found to have asymptomatic hypercalcemia on routine follow-up during the consolidation phase. Both medications were stopped. Subsequently, she was admitted to the ward and managed conservatively with hydration. Serum calcium levels were returned to normal within six days after stopping the combination of ATRA and voriconazole. We underscore that the use of anti-fungal medications should be limited while using ATRA. However, strict monitoring must be done when a combination of these drugs is started, if necessary.

## Introduction

Acute promyelocytic leukemia (APL) is a hematological malignancy, morphologically categorized as a distinct subtype of acute myeloid leukemia (M3-AML) and cytogenetically associated with a balanced translocation of the retinoic acid receptor alpha (RARα at 17q21) and the PML gene at 15q21 [1]. The introduction of all-trans retinoic acid (ATRA) as the mainstay of treatment during induction chemotherapy has revolutionized the course of disease progression with a significant decrease in disease-related mortality [2]. ATRA has a dramatic anti-tumor effect characterized by differentiation of immature promyelocytes into mature granulocytes and apoptosis of leukemic cells, followed by restoration of normal hematopoiesis as the patient enters remission. The drug is metabolized in the liver by cytochrome P-450 [3] and drug toxicity can be potentiated by inhibition of cytochrome P-450, which is known to be caused by certain drugs. The notable adverse reactions of ATRA include retinoic acid syndrome, pseudotumor-cerebri, and hypercalcemia. This case highlights an uncommon but important side-effect of ATRA thought to be due to an interaction with voriconazole and the awareness of limiting anti-fungal medications' usage simultaneously with ATRA in combined treatments. 

## Case presentation

**Figure 1:**
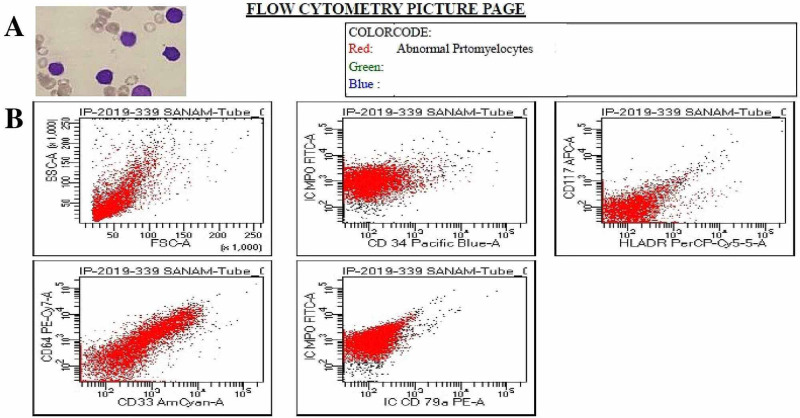
A. Peripheral blood smear shows abnormal promyelocytes. B. Flow cytometry analysis shows blasts/abnormal promyelocytes exhibiting the following phenotype; Intracytoplasmic Myeloperoxidase (+), CD33 (Positive in 21.7% of myeloid cells), CD64 (Positive in 22.1% of myeloid cells). CD33, an acute myeloid leukemia marker; CD64, a diagnostic marker of infection and sepsis.

We report the case of a nine-year-old girl who presented to the Pediatric Oncology Emergency Department at The Indus Hospital, Karachi, Pakistan in December 2019 with a two-week history of fever, decreased vision, hematemesis, and gum bleed. She was vitally unstable with a blood pressure of 90/60 mmHg and a heart rate of 120 beats per minute. Her general physical exam revealed anemia and active bleeding from gums. Systemic examination was unremarkable. Initial complete blood count (CBC) showed bicytopenia with hemoglobin of 3.4 gm/dL, platelet count of 11 x 10^9^/L, and a high leukocyte count of 30.5 x 10^9^/L, while 74% blast cells with abnormal promyelocytes were identified on a peripheral blood smear (Figure 1A).

**Figure 2:**
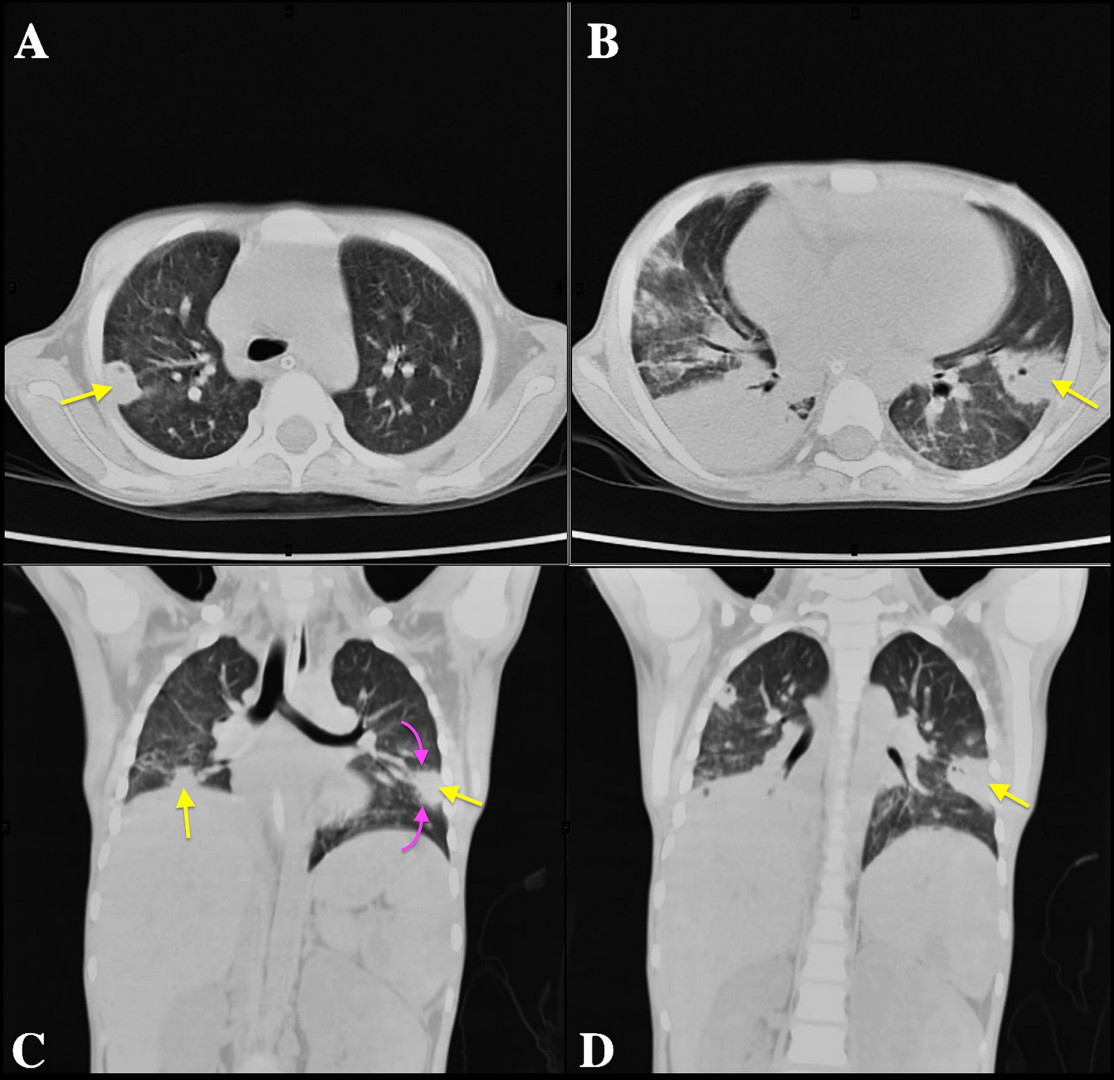
Computed Tomography (CT) of the chest without intravenous contrast demonstrate multiple peripheral nodular consolidations (yellow arrows) on transaxial (A and B) and coronal (C and D) lung windows suggestive of disseminated infection. Some of these nodules show internal cavitation indicative of necrosis. Few of them demonstrate surrounding ground-glass related to perilesional hemorrhage (purple arrows). In a patient undergoing chemotherapy, the favored diagnosis was an angioinvasive disseminated mycosis, and treatment with voriconazole was instituted.

Other laboratory findings, including coagulation profile, renal and liver function tests, serum calcium, and magnesium levels were within the normal range. Considering her irregular blood smear, flow cytometry was ordered which helped establish the diagnosis of acute promyelocytic leukemia (Figure 1B), followed by the detection of typical PML/RARα fusion gene on cytogenetics. A combination of ATRA (a dose of 45 mg/m^2^ divided in 12 hours), arsenic trioxide (ATO; 0.15 mg/kg), and daunorubicin (42 mg/m^2^) was started as induction chemotherapy (day one). She was monitored for retinoic acid syndrome and prednisolone 1 mg/kg/day was given prophylactically. ATRA was stopped on the 15th day when she started having fever spikes. Blood and urine cultures were sent which showed no bacterial growth. Further investigations were performed when her fever did not subside despite appropriate medications including meropenem, vancomycin, omeprazole, and IV hydration. To rule out causes of chest infection, gastric lavage for acid-fast bacilli (AFB) smear, culture, and gene Xpert was sent which showed no growth of mycobacterium tuberculosis. Non-contrast computed tomography (CT) scan of the chest was also done which suggested extensive disseminated fungal/granulomatous infection (Figure 2).

Elevated levels of serum (1-3)-β-D-Glucan (BDG) and galactomannan helped in confirming the diagnosis of invasive fungal chest infection. On day 23, voriconazole was commenced as an anti-fungal cover intravenously. During her further stay at the hospital, her clinical condition deteriorated, and she was intubated for assisted ventilation. However, she responded well to anti-fungal and supportive treatment. Her condition improved, chemotherapy was resumed and she was discharged after two weeks. Voriconazole was continued in addition to her chemotherapy.

**Figure 3:**
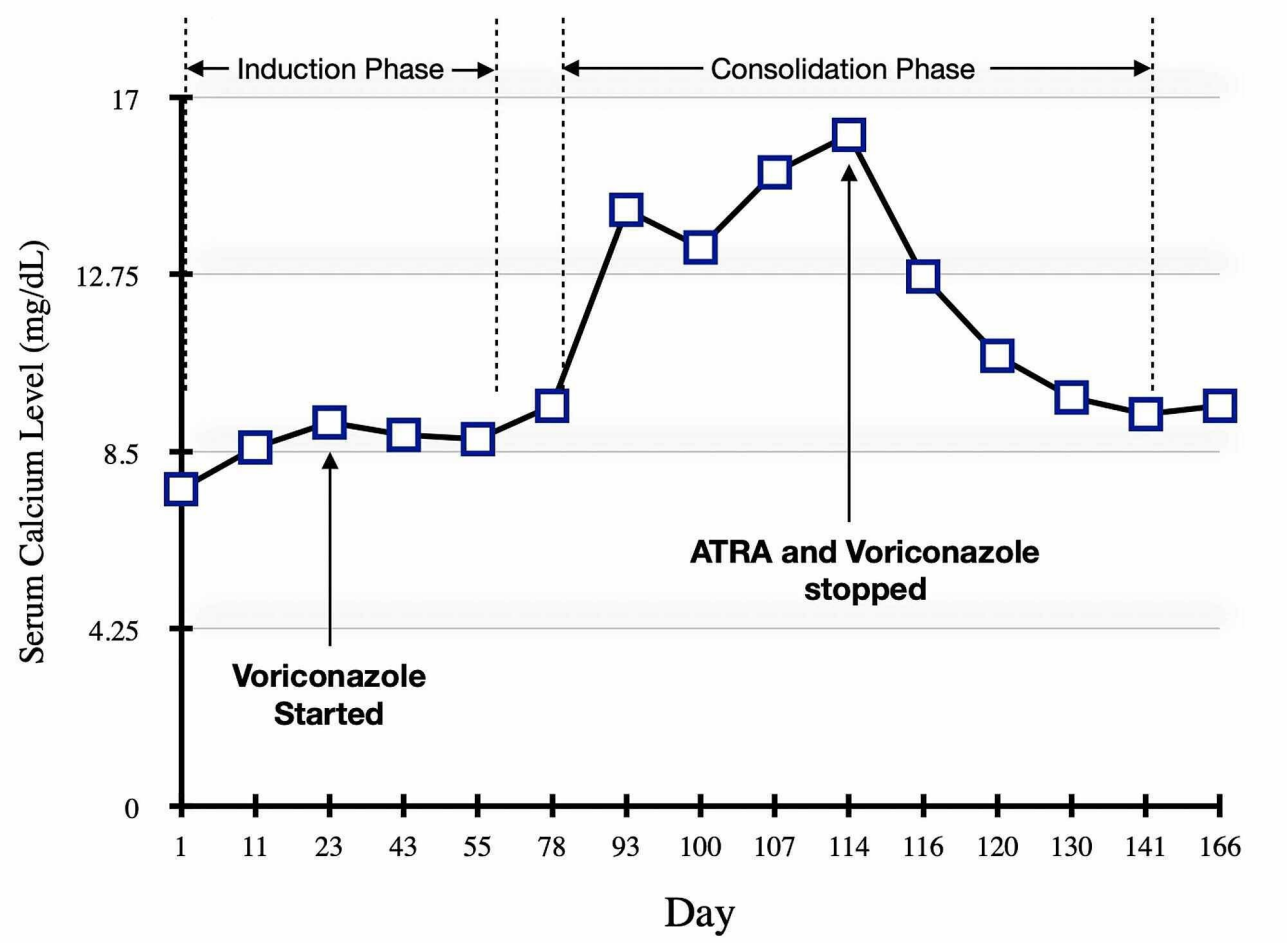
The graph illustrates serum calcium levels during two phases of chemotherapy in relation to ATRA and Voriconazole. ATRA= All-trans retinoic acid

She was followed up in the outpatient department after being discharged. Induction chemotherapy ended on day 61 and the consolidation phase with a high-risk protocol was started on day 79. The serum calcium concentration remained within normal limits until day 78. When measured again on day 93, marked elevation in serum calcium was noted (n=14.36 mg/dL) as indicated in Figure 3. 

The patient was kept on close follow-up and serum calcium levels were observed weekly. However, due to persistent asymptomatic hypercalcemia for three weeks, she was admitted again on day 114 when serum calcium levels were found to be very high (n=16.19 mg/dL). No symptoms of hypercalcemia including constipation, abdominal pain, flank pain, polydipsia, confusion, or disorientation were noticed. Her renal function tests were normal so no significant effect of hypercalcemia on kidneys was noticed. Management of hypercalcemia was done using high-volume crystalloid infusion and furosemide while ATRA and voriconazole were both stopped as together they could have been a cause of the increased serum calcium level. Work-up using available resources was done to rule out other causes of hypercalcemia including parathormone (PTH), spot serum calcium, and creatinine, which were normal, while severe vitamin D deficiency was noted (n=9.7 ng/mL). Her elevated serum calcium levels dropped on day 120, six days after stopping the combination of ATRA and voriconazole, and she was discharged after being stabilized. 

Chemotherapy was continued with arsenic trioxide until the consolidation phase was completed while ATRA was resumed to catch up for missed doses. No incidence of hypercalcemia was observed during this period after voriconazole was stopped. Currently, she is on maintenance therapy with oral methotrexate, mercaptopurine, and ATRA as molecular remission was achieved six months following induction chemotherapy.

## Discussion

All-trans retinoic acid (ATRA) is a physiological metabolite of vitamin A and is also classified as tretinoin or retinoid. Despite its exceptional results in the treatment of acute promyelocytic leukemia, it offers its own set of side effects. Hypercalcemia following the commencement of ATRA has been described on several occasions. It is thought to be either due to the direct effect of the drug on the bone osteoclastic activity leading to mineral resorption, increased serum levels of cytokines such as interleukin-6 enhancing bone resorption, or via an increased level of parathormone-related peptide (PTH-rP) [4]. Another proposed mechanism implicates inhibition of cytochrome P450 during its metabolism in the liver, subtypes CYP2C9 and CYP3A4 being the chief enzymes involved. Various drugs modulate these enzymes in an inhibitory fashion, including triazole and imidazole anti-fungal medications, reinforcing the effect of ATRA on calcium metabolism [5]. Several adverse reactions have been reported during concomitant use of ATRA with fluconazole, ketoconazole, and voriconazole resulting in pseudo-tumor cerebri, elevated serum ATRA levels, and hypercalcemia, respectively [6-8]. However, such a case has never been previously reported and documented in our city. 

We believe that the cause of hypercalcemia in this patient was due to the distinctive pharmacokinetic interaction of voriconazole with the metabolism of ATRA. To confirm the diagnosis of a presumed adverse reaction of these drugs, we noticed a sequence of events from the commencement of voriconazole following administration of ATRA and observed clinical improvement after both medications were held back. Yanamandra et al. [4] likewise discussed the possibilities of interaction of ATRA with anti-fungal therapy resulting in hypercalcemia. Based on inconclusive clinical examination, negative laboratory findings, and ongoing illness, we could not deduce any other clinical or pharmacological cause as an explanation for hypercalcemia in this patient. 

A further literature review suggests that bisphosphonates such as zoledronic acid can help manage hypercalcemia [9]. Early introduction of bisphosphonate therapy has been suggested to be the new standard of management of hypercalcemia [10]; however, due to availability and affordability issues, this drug was not considered. It has been proposed that bisphosphonates can also be started prophylactically to prevent adverse events during subsequent sessions of chemotherapy without reducing the dose of ATRA [11].

## Conclusions

The addition of all-trans retinoic acid to traditional chemotherapy regimens has dramatically improved the odds in the treatment of acute promyelocytic leukemia. However, a few adverse effects have been observed in patients receiving this drug - hypercalcemia being a rare and important side effect reported. Various medications inhibit cytochrome P450 in the liver, affecting the metabolism of all-trans retinoic acid and thus contributing to drug toxicity. For instance, voriconazole, an antifungal medication, antagonizes the metabolism of ATRA resulting in an increased incidence of adverse reactions.

We sternly recommend restricting the use of cytochrome P450 inhibitory modulators, such as azole antifungal drugs, in patients undergoing ATRA therapy to limit the incidence of hypercalcemia as a side-effect. Strict checks for hypercalcemia should be done when any of these medications are used in combination with ATRA. Based on our observations, we suggest that in addition to conventional management strategies for hypercalcemia, prompt cessation of ATRA, as well as any offending agent, should be considered to improve the outcome.
